# Protein Targeting Into the Thylakoid Membrane Through Different Pathways

**DOI:** 10.3389/fphys.2021.802057

**Published:** 2022-01-12

**Authors:** Dan Zhu, Haibo Xiong, Jianghao Wu, Canhui Zheng, Dandan Lu, Lixin Zhang, Xiumei Xu

**Affiliations:** State Key Laboratory of Crop Stress Adaptation and Improvement, School of Life Sciences, Henan University, Kaifeng, China

**Keywords:** chloroplast protein import, cpSRP pathway, cpGet pathway, spontaneous pathway, cotranslational protein transport

## Abstract

In higher plants, chloroplasts are essential semi-autonomous organelles with complex compartments. As part of these sub-organellar compartments, the sheet-like thylakoid membranes contain abundant light-absorbing chlorophylls bound to the light-harvesting proteins and to some of the reaction center proteins. About half of the thylakoid membrane proteins are encoded by nuclear genes and synthesized in the cytosol as precursors before being imported into the chloroplast. After translocation across the chloroplast envelope by the Toc/Tic system, these proteins are subsequently inserted into or translocated across the thylakoid membranes through distinct pathways. The other half of thylakoid proteins are encoded by the chloroplast genome, synthesized in the stroma and integrated into the thylakoid through a cotranslational process. Much progress has been made in identification and functional characterization of new factors involved in protein targeting into the thylakoids, and new insights into this process have been gained. In this review, we introduce the distinct transport systems mediating the translocation of substrate proteins from chloroplast stroma to the thylakoid membrane, and present the recent advances in the identification of novel components mediating these pathways. Finally, we raise some unanswered questions involved in the targeting of chloroplast proteins into the thylakoid membrane, along with perspectives for future research.

## Introduction

Chloroplasts of higher plants, the semi-autonomous organelles with internal sheet-like membrane-bound structure, are not only the sites for photosynthesis, but also for the synthesis of many essential metabolites, such as fatty acids, amino acids, vitamins, tetrapyrroles, and some phytohormones (Neuhaus and Emes, 2000; Wicke et al., 2011). Chloroplasts are believed to have evolved from an ancient cyanobacteria-like endosymbiont. Following this endosymbiosis most of the endosymbiont genes were transferred to the host nuclear genome. As a result, ∼3,000 chloroplast proteins are encoded by nuclear genes, while only ∼100 proteins are encoded by the chloroplast genome and synthesized in the stroma (Lee et al., 2017; Sjuts et al., 2017; Nakai, 2018; Richardson and Schnell, 2020). Chloroplasts contain three distinct membranes: outer envelope, inner envelope, and thylakoid membranes, which separate the chloroplast into three compartments: the intermembrane space, chloroplast stroma and thylakoid lumen (Staehelin, 2003; Kirchhoff, 2019). The thylakoid contains several photosynthetic complexes including photosystem I (PSI), photosystem II (PSII), the cytochrome *b*_6_*f* complex (Cyt *b*_6_*f*), and ATP synthase (ATPase), which are responsible for the primary reactions of photosynthesis. About half of the subunits of these complexes are encoded by the nuclear genome, synthesized in the cytosol, imported into chloroplasts and transported to the thylakoids. However, the complex structure of chloroplasts makes translocation of these proteins across the membrane system for correct targeting to their specific sub-compartment highly challenging.

**FIGURE 1:**
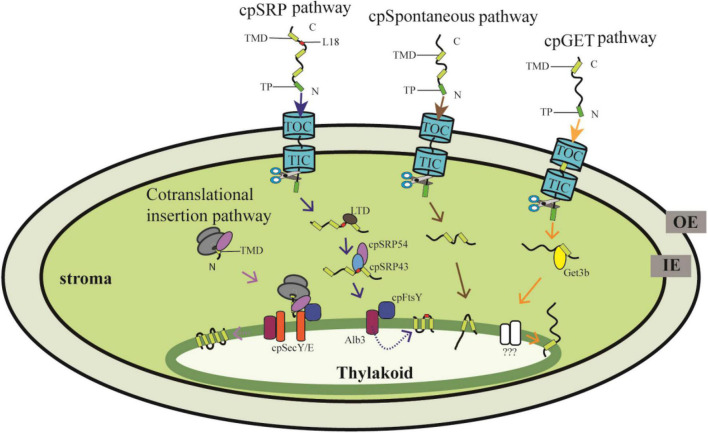
Proteins targeting into thylakoid membranes through distinct pathways. The nuclear-encoded preprotein with an N-terminal transit peptide (TP) is translocated into the chloroplast stroma through the Toc/Tic system, and then the TP is cleaved by stromal processing peptidase. The thylakoid membrane proteins are further targeted mainly through the cpSRP pathway, cpGET pathway and the spontaneous pathway. In the cpSRP-dependent pathway, LHCP is transferred from the Toc/Tic system to the SRP43/SRP54 complex with the help of LTD., cpSRP43 interacts with the L18 region between the second and third TMD of LHCP. Then the cpSRP/LHCP complex interacts with cpFtsY and finally LHCP is insert into the thylakoid membrane by the integrase Alb3. In the cpGET pathway, imported TA proteins are transferred to the targeting factor Get3b, followed by transferr to the unknown integrases. Some proteins are translocated into thylakoid membranes spontaneously. On the other hand, the chloroplast-encoded proteins are synthesized in the stroma, and cotranslationally integrated into the thylakoid membrane with the requirement of cpSec and cpSRP components. OE, outer chloroplast envelope; IE, inner chloroplast envelope. TP, transit peptide. TMD, transmembrane domain.

The nuclear-encoded thylakoid proteins with an N-terminal transit peptide (TP) are first imported into the stroma by the Toc/Tic (translocon at the outer/inner envelope membrane of chloroplasts) complexes, and then the TP is removed by the stromal processing peptidase, and the proteins are targeted into or across the thylakoid membranes through sophisticated targeting pathways (Martin et al., 2002; Leister, 2003). To date, several independent pathways have been reported, of which the chloroplast signal recognition particle (cpSRP) pathway, the spontaneous insertion pathway and the recently discovered chloroplast Guided Entry of TA proteins (cpGET) pathway are responsible for inserting proteins into the thylakoid membrane (Figure 1), while the chloroplast secretory (cpSec) and the chloroplast twin-arginine translocase (cpTat) pathways are needed for translocating proteins into the thylakoid lumen (Schünemann, 2007; Ouyang et al., 2020; Anderson et al., 2021; Xu et al., 2021a,b). In contrast, proteins encoded by the chloroplast genome are cotranslationally transported from the stroma to thylakoids (Figure 1). Although several translocation events can be distinguished through their components and energy requirements, how the imported chloroplast proteins are recognized and sorted exactly to their distinct pathways is still largely unknown. In this review, we describe the distinct transport mechanisms for targeting nuclear- and chloroplast-encoded proteins from the stroma to the thylakoid membrane. Moreover, we review recent advances and the discovery of novel components of these transport systems. Finally, we discuss current challenges and present new perspectives in this promising field.

## Pathways for Inserting the Nuclear-Encoded Proteins Into the Thylakoid Membrane

The thylakoid membrane harbors the photosynthetic apparatus composed of PSI, PSII, Cyt *b*_6_*f*, and ATP synthase. Besides, several proteins involved in thylakoid biogenesis and homeostasis are also anchored in the thylakoid membrane. These proteins are encoded by both the nuclear and chloroplast genomes, of which the nuclear-encoded proteins are translocated into thylakoid membranes through the cpSRP pathway, the spontaneous insertion pathway, and the cpGET pathway, whereas chloroplast-encoded proteins are inserted into the thylakoid membrane cotranslationally (Figure 1).

### Posttranslational Insertion Into the Thylakoid Membrane Through the Chloroplast Signal Recognition Particle Pathway

The classical SRP system is responsible for cotranslationally delivering newly synthesized proteins to their correct cellular membranes. The functional core of cytosolic SRP is composed of the SRP54 GTPase and SRP RNA, which is tightly bound to SRP54. Notably, the cpSRP system in green plants is different from all other SRP systems because the cpSRP lacks the associated RNA and instead consists of a conserved cpSRP54 and a unique chloroplast-specific cpSRP43 subunit. Contrary to the universally conserved cytosolic SRP system which only binds to the short nascent polypeptide chains and functions cotranslationally, the cpSRP system evolved posttranslational activity to target the light-harvesting chlorophyll a/b binding proteins (LHCPs) from the stroma to the thylakoid membrane (Ziehe et al., 2017, 2018; Costa et al., 2018). LHCP membrane integration requires the cpSRP43/cpSRP54 complex and its receptor cpFtsY, the integral translocase Alb3, and GTP, which is hydrolyzed by the cpSRP54 and cpFtsY GTPases (Moore et al., 2003; Ziehe et al., 2018; Figure 1).

It was first confirmed that the L18 region between the second and third transmembrane domain (TMD) of LHCP is the binding site for cpSRP43 (DeLille et al., 2000; Tu et al., 2000; Stengel et al., 2008). Recently, however, McAvoy et al. (2018) found that cpSRP43 makes more extensive interactions with all the TMDs within LHCP, thus protecting the hydrophobic LHCP from aggregation in the stroma. These new interaction sites are important for the chaperone activity of cpSRP43 based on the result that a class of cpSRP43 mutants are specifically deficient in their ability of chaperoning full-length LHCP but are not affected in their association activity with the L18 region. NMR analysis showed three conformations of cpSRP43. Interaction with cpSRP54 produces the active state through structural rearrangement, thereby improving the substrate binding efficiency of cpSRP43 (Gao et al., 2015; Liang et al., 2016). The assembly of cpSRP54 to the cpSRP43/LHCP complex leads to a much smaller LHCP/cpSRP transit complex (∼170 kDa) than the cpSRP43/LHCP complex (∼450 kDa), most likely to enable efficient LHCP insertion (Dünschede et al., 2015).

**FIGURE 2:**
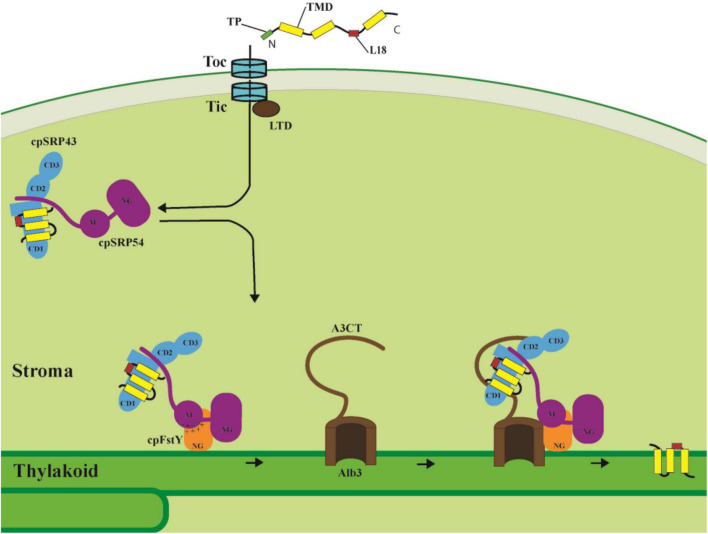
LHCPs targeting into thylakoid membranes through the cpSRP pathway. After import into the stroma through the Toc/Tic system, the LHCPs are transferred to the cpSRP43/cpSRP54 complex with the help of LTD. The L18 region between the second and third TMD of LHCP is the binding site for cpSRP43. Then cpSRP54 interacts with cpFtsY through their homologous NG domains, while the negatively charged cluster of cpSRP54 M domain interacts with the positively charged cluster of cpFtsY G domain and enhances the cpSRP54/cpFtsY complex assembly. Finally, Alb3 C-terminal tail (A3CT) interacts with the C-terminal chromodomains CD2 and CD3 of cpSRP43, and thus facilitates LHCP delivery to the Alb3 translocase and insertion into the thylakoid membrane. TP, transit peptide. TMD, transmembrane domain.

Upon formation of the cpSRP/LHCP transit complex, it is guided to the thylakoid membrane and docks to the membrane-bound cpSRP receptor cpFtsY, and then transfers LHCP to the integrase Alb3 for insertion into the thylakoid membrane. cpFtsY and cpSRP54 form a complex by association of their homologous NG domains (the N-terminal four-helix bundle and the GTPase domain), and stabilization of the cpSRP54/cpFtsY complex is dramatically activated by anionic phospholipids and the Alb3 translocase (Chandrasekar and Shan, 2017). Moreover, a new interaction site between a positively charged cluster within the cpFtsY G-domain (the GTPase domain) and a negatively charged cluster in the cpSRP54 M-domain has been identified, in which the M domain of cpSRP54 plays a similar role as the classical SRP RNA to enhance the cpSRP54/cpFtsY complex assembly (Chandrasekar et al., 2017; Figure 2).

The recruitment of the transit complex to the Alb3 translocase for LHCP insertion is mediated through the cpSRP43/Alb3 interaction. The linear motif in the Alb3 C-terminal tail (A3CT) interacts with the C-terminal chromodomains CD2 and CD3 of cpSRP43, which enables the efficient delivery of LHCP to the Alb3 integrase (Falk et al., 2010; Horn et al., 2015; Figure 2). A novel model has been proposed in which the conformational dynamics of cpSRP43 enables LHCP capture and release. Distinct conformations of cpSRP43 allow it to be activated by cpSRP54 assembly in the stroma to capture LHCP and inactivated by association with Alb3 translocase in the thylakoid membrane to release LHCP (Liang et al., 2016). Interestingly, cpSRP43 also directly interacts with the N-terminal of glutamyl-tRNA reductase (GluTR), a rate-limiting enzyme in tetrapyrrole biosynthesis (TBS), chaperones and stabilizes GluTR, and consequently optimizes chlorophyll biosynthesis. This interaction between cpSRP43 and GluTR reveals a posttranslational coordination for LHCP insertion with chlorophyll biosynthesis (Wang et al., 2018). More strikingly, high temperature drives the dissociation of the cpSRP43/54 complex, thus freeing cpSRP43 to interact with GluTR, CHLH (Mg-chelatase H subunit) and GUN4 (Genomes uncoupled 4) to protect them from heat-induced aggregation (Ji et al., 2021). Taken together, cpSRP43 not only functions as the hub for LHCP membrane insertion since it recruits cpSRP54, chaperones LHCP, and provides the docking site for Alb3 translocase, but also chaperones proteins for chlorophyll biosynthesis and thus coordinates the assembly of chlorophyll and LHCP into the light-harvesting complex.

Several new components of the cpSRP pathway have also been identified. An *Arabidopsis* ankyrin protein, LTD., which is located in the stroma and interacts with the Tic complex, LHCP and cpSRP43, was demonstrated to handover LHCP from the Tic translocon to the cpSRP43/cpSRP54 complex (Ouyang et al., 2011). In addition, *STIC1* (suppressor of *tic40*) and *STIC2* were revealed to act in the cpSRP54/cpFtsY-involved transport pathway. The *STIC1* gene encodes the Alb3 paralog protein, Alb4, while *STIC2* encodes a novel stromal protein that associates with Alb3 and Alb4. Loss of STIC1/Alb4 and STIC2 proteins may mitigate the defect of protein import caused by the *TIC40* mutation, thus revealing a potential link between the envelope and thylakoid protein transport systems (Bédard et al., 2017).

### Direct Insertion Into the Thylakoid Membrane Through the Spontaneous Pathway

Many proteins such as the photosystem subunit PsaG, PsaK, PsbW, PsbX, and PsbY, the ATPase subunit CFo-II, and the cpTat translocase subunits Tha4 and Hcf106, are integrated into the thylakoid membrane *via* the spontaneous insertion pathway which does not need any energy or proteinaceous components. This direct insertion pathway was first proposed for CFo-II, a nuclear-encoded single-membrane-spanning component of the ATP synthase. Treatments that disrupt the cpSRP, cpSec and cpTat dependent pathways do not affect the integration of CFo-II, suggesting a direct interaction of CFo-II with the lipid components of the membrane (Michl et al., 1994; Robinson et al., 1996). CFo-II, PsbW, and PsbX all span the thylakoid membrane with a single hydrophobic domain, whereas PsaG and PsaK insert into the membrane with two transmembrane spans (Lorkovic et al., 1995; Kim et al., 1998; Thompson et al., 1998; Mant et al., 2001; Zygadlo et al., 2006; Aldridge et al., 2009). For PsbY, the two transmembrane spans partition into the lipid bilayer of the thylakoid membrane and provide the driving force required for the translocation of the intervening charged region through hydrophobic interactions (Thompson et al., 1999).

The mechanism of insertion of the M13 procoat protein into the cytoplasmic membrane of *E. coli* was also originally proposed to be by the spontaneous pathway. However, it was subsequently found that YidC, the homolog of the chloroplast Alb3 insertase, is actually required for the insertion of the M13 procoat protein, which raised the question if there exists a truly spontaneous insertion pathway (Kuhn et al., 1986; Samuelson et al., 2000). PsbW and PsbX have remarkable structural similarities with the M13 procoat protein. However, inactivation of Alb3 did not affect PsbW and PsbX insertion into the thylakoid membrane, suggesting a direct insertion pathway for these proteins (Woolhead et al., 2001).

### Insertion of Tail-Anchored Proteins Into the Thylakoid Membrane Through the Chloroplast Guided Entry of TA Proteins Pathway

In a typical eukaryote cell, 3%∼5% of the membrane proteins are tail-anchored (TA) proteins with the feature of a cytosolic-facing N-terminal domain and a single C-terminal TMD followed by a tail within 30 amino acids. In these proteins TMD acts both as a membrane anchor and a targeting signal (Shigemitsu et al., 2016; Mateja and Keenan, 2018). TA proteins are found in all cellular membranes and play key roles in protein translocation, membrane fusion, vesicular trafficking, apoptosis, organelle biogenesis, and other essential cellular processes (Denic et al., 2013).

**FIGURE 3:**
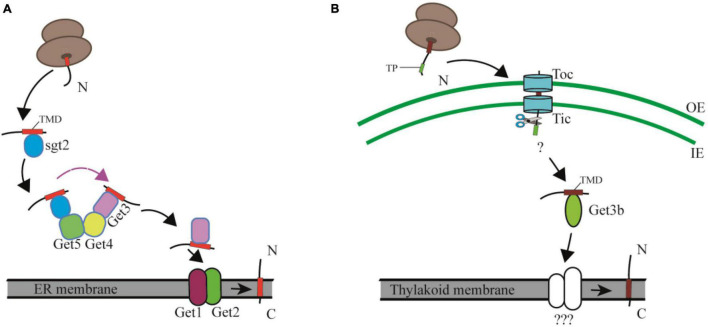
Comparison of the cytosolic and chloroplast GET pathway. **(A)** In the cytosolic GET pathway, the newly synthesized TA proteins are firstly captured by Sgt2, and then transferred to the targeting factor Get3 by the Get4/Get5 scaffold complex, and finally integrated into ER membranes with the facilitation of Get1 and Get2. TMD, transmembrane domain. **(B)** In the cpGET pathway, imported TA proteins are transferred to the targeting factor Get3b, and then integrated into the thylakoid membrane with the aid of unknown integrases. OE, outer chloroplast envelope; IE, inner chloroplast envelope. TP, transit peptide. TMD, transmembrane domain.

The posttranslational insertion of TA proteins from the cytosol to cellular membranes depends on the GET pathway in yeast and the TRC (Transmembrane Recognition Complex) pathway in mammals (Farkas et al., 2019; Farkas and Bohnsack, 2021). The yeast cytosolic GET machinery includes a pretargeting complex consisting of Sgt2, a small glutamine-rich tetratricopeptide repeat (TPR)-containing protein (SGTA in mammals), Get4 (TRC35 in mammals), and Get5 (UBL4 in mammals), of which Sgt2 captures newly synthesized TA proteins from the ribosomal exit tunnel and is considered the most upstream factor in the GET pathway (Wang et al., 2010; Shao et al., 2017; Zhang et al., 2021). Subsequently Get4 and Get5 build up a “scaffolding complex” by recruiting Get3 and the Sgt2-TA complex, respectively. This scaffolding complex prevents aggregation and promotes loading of TA proteins onto Get3 (Bat3 in mammals), the central targeting factor that forms a closed structure after binding ATP. The resulting hydrophobic groove recognizes the TMD of TA proteins. At the endoplasmic reticulum (ER) membrane, Get2 (CAML in mammals) aids Get1 (WRB in mammals) to form a tight complex with nucleotide-free Get3, resulting in the release of the TA protein for insertion into the membranes. Finally, Get3 is recycled to the cytosol to initiate a new round of targeting (Schuldiner et al., 2008; Gristick et al., 2014; Rome et al., 2014; Wang et al., 2014; Rao et al., 2016; Figure 3A). A recent study showed that the cytoplasmic helix α3′ of Get2/CAML forms a “gating” interaction with Get3/TRC40, which is important for guiding TA proteins insertion into membranes (McDowell et al., 2020).

While the GET pathway was originally identified in mammals and yeasts, it was recently found to be partially conserved in higher plants as well. Orthologs of Get1, Get3, Get4, Get5, and Sgt2, but not Get2 have been identified in plant genomes through bioinformatic approaches *Get1*, *Get3*, *Get4*, *Get5*, and *Sgt2*, *Get2* (Paul et al., 2013; Srivastava et al., 2017). Further phylogenetic analysis of 18 species revealed two Get3 clades termed Get3a and Get3bc, of which *At*Get3a, *At*Get3b, and *At*Get3c are localized in the cytosol, chloroplast and mitochondria, respectively. Loss of *AtGet1* or *AtGet3a* results in a significant reduction of the root hair-specific protein SYP123, and thus leads to root hair growth defects in these *Atget* mutant lines (Xing et al., 2017). Most surprisingly, overexpression of *AtGet3a* in the *Atget1* mutant causes a more severe phenotype in root, silique and seed development compared with the parental lines. The absence of GET pathway components may trigger alternative insertion pathways. However, overexpression of *AtGet3a* in the receptor mutant *Atget1* might cause cytosolic *At*Get3a/TA protein aggregates and a consequent TA protein insertion block through the alternative insertion pathway (Xing et al., 2017).

Recently, *Arabidopsis* Get2 was also discovered by an immunoprecipitation-mass spectrometry (IP-MS) method. Using the *AtGet1-GFP* transgenic plants, G1IP (*At*Get1-interacting protein) was identified. G1IP exhibits low sequence similarity but high structural similarity to Get2/CAML (Asseck et al., 2021). Both G1IP and *At*Get1 show a subcellular ER localization and share the same expression profile. The *g1ip* mutant shows reduced root hair elongation, which is similar to other *Atget* lines. Moreover, G1IP interacts with *At*Get1 and *At*Get3a, and expression of G1IP and *At*Get1 together can complement the yeast GET receptor mutant Δ*get1get2* strain. Besides, the G1IP N terminus contains a conserved cluster of positively charged amino acids which is essential for the binding of Get3/TRC40, and alteration of this motif in G1IP is sufficient to inhibit TA protein transport. These results strongly suggest that *Arabidopsis* G1IP encodes the functional ortholog of Get2, although only a small part of its sequence is conserved between yeasts, plants and mammals (Asseck et al., 2021).

Based on the facts that only Get3b and Get3c, but no other Get system components are found in organelles, and that Get3bc cannot rescue the growth defect of the *Atget3a* mutant, it was previously considered to be unlikely that the organellar Get3 homologs were involved in TA protein insertion (Xing et al., 2017). However, Anderson et al. recently demonstrated that *At*Get3b is structurally similar to the cytosolic Get3 protein and serves as a targeting factor to deliver TA proteins from the chloroplast stroma to the thylakoid membrane (Anderson et al., 2021; Figure 3B). Chloroplast membranes contain several TA proteins, such as cpSecE1, a component of the thylakoid Sec1 translocase and cpSecE2, a component of the chloroplast inner envelope Sec2 translocase (Schüenemann et al., 1999; Li et al., 2015). Both *in vitro* and *in vivo* assays indicated that *At*Get3b interacts with cpSecE1 rather than cpSecE2 to form a homodimer or oligomer in the chloroplast stroma, mediated by the C-X-X-C zinc coordination motif of *At*Get3b (Anderson et al., 2021). A domain-swapping strategy used between cpSecE1 and cpSecE2 suggests that the TMDs and C-terminal tails of TA proteins are key factors that specify the interaction between *At*Get3b and its client TA proteins (Anderson et al., 2019). The physicochemical features of the TMD and C-terminal regions of cpSecE2 make it incompatible with the hydrophobic groove formed by *At*Get3b oligomerization (Anderson et al., 2021).

Loss of *get3b* in *Arabidopsis* displays a visually indistinguishable phenotype from the wild type. Similar observations have been made with the yeast *get3* mutant, suggesting that the Get system may operate in parallel or at least be partially redundant with other insertion pathways (Anderson et al., 2021). Consistent with this notion, the *get3bsrp54* double mutant and the *get3bsrp43srp54* triple mutant show much more severe defects in growth, including pronounced dwarfism, obvious chlorosis and dramatically reduced PSI and PSII activities. These results demonstrate synergistic interactions between cpGET and the cpSRP pathways in delivering proteins into thylakoid membranes (Anderson et al., 2021).

## Cotranslational Insertion of the Chloroplast-Encoded Proteins Into the Thylakoid Membrane

Chloroplast genes encode ∼37 thylakoid membrane proteins such as the PSII reaction center protein D1 and D2, and the PSI subunits PsaA and PsaB, which are synthesized in the stroma and integrated into thylakoid membranes through a cotranslational process (Zoschke and Barkan, 2015). However, little is known about the mechanisms that target these proteins into the thylakoid membrane. The *cpSRP54* (*ffc*) mutant contains much lower levels of D1, D2, PsaA, and PsaB proteins, suggesting the involvement of *cpSRP54* in the cotranslational pathway (Amin et al., 1999). Cross-linking experiments revealed an interaction between the D1-ribosome nascent chain complex and cpSRP54, but not cpSRP43 (Nilsson et al., 1999). cpSRP54 is present in two stroma pools. In one of them it is recruited by cpSRP43 to function in the posttranslational targeting for LHCPs, and in the other pool it is associated with 70S ribosomes to function in the cotranslational import of proteins such as D1 (Franklin and Hoffman, 1993; Schüenemann et al., 1998). The interaction between cpSRP54 and D1 involves the first TMD of D1, and only occurs when the D1 nascent chain is still attached to ribosomes, suggesting that cpSRP54 functions in the early steps of D1 biogenesis (Nilsson and van Wijk, 2002).

Besides cpSRP54, the SRP receptor cpFtsY is also considered to function in the cotranslational integration process of chloroplast-encoded thylakoid membrane proteins, based on the observation of a strong reduction of D1 in the *cpftsy* mutant (Tzvetkova-Chevolleau et al., 2007; Asakura et al., 2008). Zhang et al. (2001) demonstrated a direct interaction between D1 elongation intermediates and the chloroplast Sec translocon component cpSecY, suggesting that the cpSec pathway not only functions in posttranslational targeting of nuclear-encoded proteins, but also in cotranslational insertion of chloroplast-encoded proteins. The Alb3 integrase of the cpSRP pathway is also reported to act in a later insertion process, facilitate release of D1 from the cpSecY translocase, and promote the assembly of D1 into PSII (Ossenbühl et al., 2004; Walter et al., 2015). Thus, the integration of D1 into the thylakoid membrane suggests a functional link between the cpSRP and the cpSec translocation machinery. Further evidence for this speculation comes from the integration of Cytochrome f. Cytochrome f insertion into the thylakoid membrane was initially thought to be mediated through the cpSec translocon. However, further analysis showed a severely decreased level of Cytochrome f in the *cpftsy* mutant, indicating a coordinated mechanism between the cpSRP and cpSec translocation pathways (Nohara et al., 1996; Asakura et al., 2008).

Recently, Zoschke and Barkan performed a comprehensive analysis of chloroplast ribosome profiling with separated soluble and membrane fractions in maize. They found that about half of the chloroplast-encoded thylakoid membrane proteins are targeted cotranslationally while the other half is targeted posttranslationally. Synthesis of these cotranslationally integrated proteins are initiated on stromal ribosomes, and then transferred to the thylakoid-attached ribosomes in a nuclease-resistant fashion shortly after the emergence of the first transmembrane segment of the nascent peptide. In contrast, membrane proteins whose translation terminates before a transmembrane segment emerge from the ribosome’s exit channel will be integrated into thylakoid membranes posttranslationally. These results suggest a key role of the first transmembrane segment in linking ribosomes to the thylakoid membrane for cotranslational targeting (Zoschke and Barkan, 2015).

## Conclusion and Future Perspectives

In the past decades, much progress has been made in understanding the molecular details of targeting both nuclear- and chloroplast-encoded proteins into the thylakoid membranes. However, several central issues still need to be further investigated in the future. One avenue of study will be to explore novel components of these translocation pathways. During evolution, ancient translocation systems acquired and developed new proteins and mechanisms to facilitate the correct targeting of chloroplast preproteins. Thus, identification of these novel components may provide new insights into how plant cells have adapted prokaryotic mechanisms to the eukaryotic environment. Another area yet to be examined is the overall structure of these distinct translocases, a challenging task given their highly dynamic and transient nature. Furthermore, the recent discovered cpGET pathway has broadened our understanding of protein targeting into thylakoid membranes. However, only Get3b in this system has been identified, thus the search for the remaining components of the cpGET pathway constitutes an important task for the future.

## Author Contributions

DZ and XX designed and wrote the manuscript. DZ, JW, CZ, and DL collected and analyzed the data. LZ and XX revised the manuscript. HX produced the figures. All authors contributed to the article and approved the submitted version.

## Conflict of Interest

The authors declare that the research was conducted in the absence of any commercial or financial relationships that could be construed as a potential conflict of interest.

## Publisher’s Note

All claims expressed in this article are solely those of the authors and do not necessarily represent those of their affiliated organizations, or those of the publisher, the editors and the reviewers. Any product that may be evaluated in this article, or claim that may be made by its manufacturer, is not guaranteed or endorsed by the publisher.
